# Multitargeting Effects of Calebin A on Malignancy of CRC Cells in Multicellular Tumor Microenvironment

**DOI:** 10.3389/fonc.2021.650603

**Published:** 2021-09-29

**Authors:** Constanze Buhrmann, Ajaikumar B. Kunnumakkara, Aviral Kumar, Marek Samec, Peter Kubatka, Bharat B. Aggarwal, Mehdi Shakibaei

**Affiliations:** ^1^ Musculoskeletal Research Group and Tumor Biology, Chair of Vegetative Anatomy, Institute of Anatomy, Faculty of Medicine, Ludwig-Maximilian-University Munich, Munich, Germany; ^2^ Institute of Anatomy and Cell Biology, Faculty of Medicine, University of Augsburg, Augsburg, Germany; ^3^ Cancer Biology Laboratory & Department of Biotechnology-National institute of Advanced Industrial Science and Technology (DBT-AIST) International Center for Translational and Environmental Research (DAICENTER), Department of Biosciences & Bioengineering, Indian Institute of Technology Guwahati, Assam, India; ^4^ Department of Obstetrics and Gynecology, Jessenius Faculty of Medicine, Comenius University in Bratislava, Martin, Slovakia; ^5^ Department of Medical Biology, Jessenius Faculty of Medicine, Comenius University in Bratislava, Martin, Slovakia; ^6^ Inflammation Research Center, San Diego, CA, United States

**Keywords:** Calebin A, colorectal cancer, T-lymphocyte, TNF-β, stromal cell, tumor microenvironment, NF-κB

## Abstract

**Background:**

Tumor microenvironment (TME) provides the essential prerequisite niche for promoting cancer progression and metastasis. Calebin A, a component of *Curcuma longa*, has long been investigated as a safe multitargeted agent with antitumor and anti-inflammatory properties. However, the multicellular-TME-induced malignancy and the antitumorigenic potential of Calebin A on colorectal cancer (CRC) cells in 3D-alginate cultures are not yet understood, and more in-depth research is needed.

**Methods:**

3D-alginate tumor cultures (HCT116 cells) in the multicellular proinflammatory TME (fibroblast cells/T lymphocytes), tumor necrosis factor beta (TNF-β)-TME (fibroblast cells/TNF-β) were treated with/without Calebin A to address the pleiotropic actions of Calebin A in the CRC.

**Results:**

We found that Calebin A downmodulated proliferation, vitality, and migration of HCT116 cells in 3D-alginate cultures in multicellular proinflammatory TME or TNF-β-TME. In addition, Calebin A suppressed TNF-β-, similar to multicellular-TME-induced phosphorylation of nuclear factor kappa B (NF-κB) in a concentration-dependent manner. NF-κB-promoting proinflammatory mediators, associated with tumor growth and antiapoptotic molecules (i.e.,MMP-9, CXCR4, Ki-67, β1-integrin, and Caspase-3) and its translocation to the nucleus in HCT116 cells, were increased in both TME cultures. The multicellular-TME cultures further induced the survival of cancer stem cells (CSCs) (upregulation of CD133, CD44, and ALDH1). Last but not the least, Calebin A suppressed multicellular-, similar to TNF-β-TME-induced rigorous upregulation of NF-κB phosphorylation, various NF-κB-regulated gene products, CSCs activation, and survival in 3D-alginate tumor cultures.

**Conclusions:**

The downmodulation of multicellular proinflammatory-, similar to TNF-β-TME-induced CRC proliferation, survival, and migration by the multitargeting agent Calebin A could be a new therapeutic strategy to suppress inflammation and CRC tumorigenesis.

## Introduction

The increasing incidence of colorectal cancer (CRC) worldwide combined with high mortality rates necessitate the eminent need to develop newer therapeutic strategies to regress the current negative trend in global CRC statistics (1). CRC is a multifactorial heterogeneous disease, as environmental factors influencing lifestyle (diet, smoking, and alcohol) and a genetic predisposition interact and contribute to its development. In CRC, the multistage process from a benign polyp to an invasive adenocarcinoma is supported with a long-lasting chronic low-grade inflammation that promotes the development of malignant cells (2, 3). In fact, it has been reported that patients with chronic inflammatory diseases of the colon, such as chronic inflammatory bowel disease, have an increased risk of CRC (4). It is now widely accepted that cancer’s malignant progression is mediated by the crosstalk between tumor cells and the surrounding stromal cells (5–7). Under the influence of cytokines, growth factors, and chemotactic stimuli, the cancer cells recruit the stromal fibroblasts and transform them, leading to a rearrangement of the stromal extracellular matrix (ECM) and creating a localized stimulating tumor microenvironment (TME) (5, 8).

The TME is multicellular and consists of a highly complex interaction between the tumor cells, stromal cells, immune cells, and microorganisms (9). This multicellular interaction, based on soluble factors, transformed ECM, epigenetic modifications, immune cells, and transformed fibroblasts, not only induces the malignancy and metastasis of tumors but also delays and blocks the response to tumor treatment leading to the acquisition of drug resistance (9). Tumor-recruited fibroblasts, also known as cancer-associated fibroblasts (CAFs), have increased migration capacity, secrete tumor-stimulating factors into the TME, and are considered key modulators in the process of ECM transformation in the TME (10). Besides, tumor-associated fibroblasts, immune cells, which have a broad spectrum of proinflammatory and anti-inflammatory functions, play an essential role in maintaining tissue homeostasis in the intestine, and their dysregulation leads to chronic inflammation, which drives cancer development (11). Immune cells such as macrophages and lymphocytes have a context-dependent, dual function in cancer progression by orchestrating an anti-neoplastic activity or a tumor-promoting tissue repair response by producing reactive oxygen species and inflammatory cytokines (12, 13). Interestingly, it has been observed that tumor-infiltrating lymphocytes exercise protumorigenic activities and help to evade an immunological response (14, 15). Therefore, the tumor-associated immune cells not only inhibit the antitumor activity but also act as prominent mediators of tumor development and metastasis (16) and could be identified as novel therapeutic targets circumventing CRC.

The transcription factor NF-κB regulates the activation of several proinflammatory and antiapoptotic genes and has been described to be constitutively expressed in metastatic CRC (17–19). Moreover, the constitutive activation of the proinflammatory NF-κB and its downstream signaling cascade in TME promotes tumor cell survival and stimulates the misdirected immunosuppressive activity of immune cells (14, 20). NF-κB induces the production and activation of proinflammatory cytokines that are prominent members of the tumor necrosis factor (TNF) family, such as TNF-α and TNF-β, also known as lymphotoxin-α (21, 22). A plethora of literature has implicated the functional role of TNF-α and TNF-β in promoting cancer development through autocrine and paracrine signal transduction involved in cancer cell proliferation, invasion, and metastasis even in CRC (22–25). Interestingly, it has been reported that lymphocytes including B- and T-cells in the TME stimulate tumor progression by secreting TNF-β (26). In addition, NF-κB-dependent transcription factors were upregulated in tumor-associated lymphocytes, which promoted proliferation/invasion and induced inflammation and T-cell depletion pathways (27). Due to the aforementioned facts, the focus on cytokine receptor pathways could be a promising antitumor strategy (16). Under physiological conditions, integrins are essential regulators of cell survival, proliferation, adhesion, and migration in all tissues by mediating cell–cell and cell–matrix interactions (28). Interestingly, integrins have been shown to play a critical role in mediating the interaction between tumor cells and TME, and drugs targeting this interaction may offer new therapeutic potential (29, 30). Colorectal cancer stem cells (CSCs) play a central role in the crosstalk between the tumor and its TME. They secrete chemokines and cytokines, which in turn trigger cancer malignancy and metastasis and could be an important target for devising new therapeutic strategies for combating CRC (31, 32).

Mono-target therapies have proven to be unsatisfactory with limited clinical outcomes because they cannot meet the challenge posed by multicellular TME. Therefore, the identification of novel multitargeting agents is imperative for effective strategies targeting not only tumor cells but, more importantly, the multicellular TME. Natural polyphenols have been studied extensively for their multitargeted, antineoplastic activities in the last decade (33, 34). Calebin A is a recently isolated pharmacologically active component of the turmeric rhizome (*Curcuma longa* L. *Zingiberaceae*), which is widely used in traditional Ayurvedic and Chinese herbal medicine (35–37). Calebin A possesses antitumor properties and has been shown to target the NF-κB signaling pathway (35, 38). Recent research has shown that Calebin A blocks TNF-β-induced activation of the NF-κB signaling pathway, thereby inhibiting proliferation, invasion, and apoptosis in CRC cells (23, 39). Furthermore, Calebin A has great potential for chemosensitizing CRC cells compared to the standard treatment with 5-fluorouracil (5-FU) (39).

Given the important role that leukocyte cells, cytokines, and stromal fibroblast cells in the TME play in promoting CRC tumorigenesis (proliferation, CSCs, and invasion), we have developed a novel 3D multicellular proinflammatory TME that combines CRC cells, T-lymphocytes, and fibroblasts *in vitro* to better mimic a heterogeneous proinflammatory TME similar to *in vivo* conditions. We hypothesized that the functional and biochemical interaction between tumor cells and stroma in this TME could induce tumor metastasis by activating the NF-κB signaling pathway, its downstream effector molecules, and CSCs. Based on our hypothesis, this interaction could be reversed by the natural polyphenol Calebin A treatment. In addition, using our progressive experimental approach and the consequent gaining of original results may help to potentially uncover newer insights and opportunities for the treatment and clinical management of CRC malignancy and extend our knowledge about mechanisms associated with CRC progression.

## Materials and Methods

### Antibodies and Chemicals

Monoclonal antibodies to p65, and phospho-specific p65-NF-κB, MMP-9, CXCR4, cleaved-Caspase-3, and β1-Integrin, were obtained from R&D Systems (Heidelberg, Germany). Antibodies to β-actin were from Sigma-Aldrich (Taufkirchen, Germany). Secondary rhodamine-coupled antibodies for immunofluorescence and anti-Ki67 were from Dianova (Hamburg, Germany), and alkaline phosphatase-linked antibodies for Western blotting were from EMD Millipore (Schwalbach, Germany). Monoclonal anti-ALDH1 was obtained from Acris Antibodies GmbH (Herold, Germany). Monoclonal anti-CD133 and anti-CD44 were purchased from Abcam PLC (Cambridge, UK). MTT reagent (3-(4,5-dimethylthiazol-2-yl)-2,5-diphenyltetrazolium bromide), 4′,6-diamidino-2-phenylindole (DAPI), and alginate were from Sigma-Aldrich (Taufkirchen, Germany). TNF-β was purchased from eBiosciences (Frankfurt, Germany). Furthermore, TNF-β was given as a kind gift by Genetech (South San Francisco, CA, USA). Calebin A was a generous gift from Sabinsa Corporation (East Windsor, NJ, USA). Calebin A was diluted as 10,000 µM stock solution in dimethyl sulfoxide (DMSO) and further diluted in cell culture medium for experimental investigations. The final concentration of DMSO did not exceed 0.1% during the experiments.

### Cells and Cell Culture Conditions

The CRC cell line HCT116 and normal human fibroblast cells (MRC-5) were obtained from the European Collection of Cell Cultures (Salisbury, UK).

HCT116 and MRC-5 cells were cultured as monolayers under standard conditions (37°C, 5% CO_2_) with whole-cell culture growth medium [10% fetal calf serum (FCS)] as previously described (40) and passaged when cells reached 70–80% confluency. A human T-lymphocyte cell line (Jurkat cells) was cultured in suspension with a whole-cell culture growth medium containing 10% FCS (41). Before all experiments, the cells were washed three times with serum-starved medium (3% FCS) and further incubated for 30 min with the same medium before initiation of experiments. All experiments were performed with the serum-starved medium.

### Study Design

**Figure 1:**
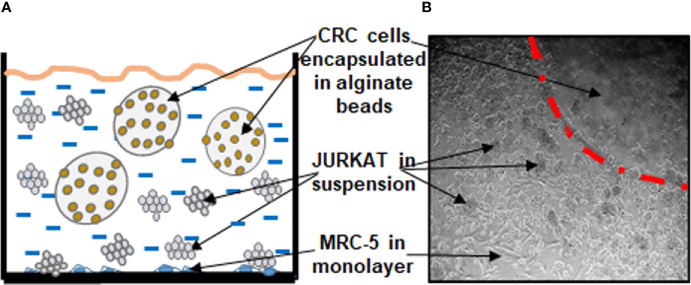
Working model showing the experimental procedures of multicellular proinflammatory TME cultures as a scheme **(A)** and light microscopic photograph **(B)**.

The aim of this study was to establish a multicellular proinflammatory TME culture *in vitro* (**Figures 1A, B**).

As “basal control,” CRC cells (HCT116) encapsulated in 3D-alginate beads, were cultured alone.

To establish the “multicellular proinflammatory TME” culture, stromal fibroblasts (MRC-5) were first seeded in monolayer (3,000 cells/cm^2^) and allowed to adhere to the bottom of the Petri dishes for 24 h in 10% FCS whole-cell culture growth medium. In the next step, CRC cells were encapsulated in 3D-alginate beads as described below. Finally, to establish the “multicellular proinflammatory TME” cultures, these HCT116-alginate beads and T-lymphocytes (Jurkat cells) (10,000/ml) were added to the Petri dishes containing the monolayer fibroblasts and all co-cultured in serum-starved medium (3% FCS).

To compare the “multicellular proinflammatory TME” and the role of proinflammatory cytokines in the TME, additional experiments were performed without T-lymphocytes but with TNF-β (10ng/ml), and these were referred to as “TNF-β-TME” cultures.

Overall, for the experiments, HCT116 cells were cultured in alginate beads either alone (basal control) or in “multicellular proinflammatory TME” culture or in “TNF-β-TME” culture alone or in combination with Calebin A in a concentration-dependent manner (1, 2, and 5 µM) for 10 days.

### Alginate Bead Culture

3D-alginate bead cultures were prepared as previously described in detail (40, 42). Briefly, CRC HCT116 cells (1 × 10^6^/ml) were resuspended in sterile liquid alginate (2% in 0.15 M NaCl, stirring for 1–2 h), and this suspension was added dropwise to a sterile CaCl_2_ solution (100 mM) where the drops polymerized to alginate beads. After polymerizing of the beads in the CaCl_2_ solution for 15 min, the beads were washed three times with a NaCl solution (0.15 M) and twice with the whole-cell culture medium. Finally, the beads were washed with serum-starved medium, incubated for 30 min with serum-starved medium, and finally transferred to Petri dishes that were either empty (for basal control) or contained MRC-5 in monolayer for creating TME cultures.

### Vitality Assay of CRC Cells From Alginate Cultures

Viability and proliferation potential of CRC HCT116 cells in the proinflammatory TME culture was evaluated by the MTT method as described in detail (25). Briefly, alginate beads were dissolved for 20 min in a sterile 55 mM sodium citrate solution to release the HCT116 cell from the alginate. After washing twice with balanced Hank’s salt solution, HCT116 cells were resuspended in modified whole-cell culture medium (without phenol red, without vitamin C, 3% FCS) and immediately distributed to a 96-well plate (100 µl cell suspension/well) and 10 μl MTT solution (5 mg/ml) was added to each well. After 3 h incubation, the reaction was blocked by adding 100 µl of MTT solubilization solution (10% Triton X-100/acidic isopropanol) to each well. After overnight incubation at 37°C, metabolically active tumor cells were determined by measuring the optical density (OD) at 550 nm (OD550) using a revelation 96-well multiscanner plate ELISA reader (Bio-Rad Laboratories Inc. Munich, Germany).

### Migration and Colony Formation Assay

CRC HCT116 cells were cultured in TME as described above, and colonosphere formation and migration capacity were investigated as previously described (23). Briefly, on day 10, colonosphere formation in the beads was quantified by counting 20 microscopic fields with a phase-contrast microscope (Zeiss, Germany), and images were digitally stored. The invasion was quantified by staining adhered colonies at the bottom of the Petri dish with toluidine blue, colonies manually counted under a light microscope (Zeiss, Germany), and images captured and digitally stored.

### Immunofluorescence Labeling

For immunofluorescence investigations, the “multicellular proinflammatory TME” culture described above was slightly modified (**Figure 4**). Stromal fibroblasts were seeded in a monolayer on the bottom of a Petri dish (3,000 cells/cm^2^), and CRC cells HCT116 were seeded separately in a monolayer on glass plates (5,000/glass plate); the cells were let to adhere for 24 h in whole-cell culture growth medium (10% FCS). To establish the “multicellular proinflammatory TME” cultures, the HCT116-containing glass plates were placed on a steel net bridge into the Petri dishes containing the fibroblast culture, and T-lymphocytes (10,000/ml) were added. To allow the development of proinflammatory TME conditions, cultures were kept in a serum-starved medium for 24 h before starting treatment (**Figure 4**). To compare the “multicellular proinflammatory TME” and the role and potential of proinflammatory cytokines in the TME, additional experiments for immunofluorescence were performed without T-lymphocytes but with TNF-β (10 ng/ml), and these were referred to as “TNF-β-TME” cultures. For “basal control,” CRC cells were cultured on glass plates (5,000/glass plate) alone. For the experiments, they were either cultured alone (basal control) or cultured in “multicellular proinflammatory TME” or in “TNF-β-TME” alone or in combination with Calebin A in a dose-dependent manner (1, 2, and 5 µM) for 4 h.

For immunofluorescence investigation, HCT116 cells were was fixated with methanol, washed in phosphate-buffered saline (PBS), and incubated overnight (4°C) with primary NF-κB antibodies [dilution 1:100 in PBS/bovine serum albumin (BSA 1%)], followed by secondary antibody incubation (rhodamine-coupled, 1:100) for 2 h. Finally, to visualize nuclei, samples were stained with DAPI for 15 min, embedded with Fluoromount (Sigma-Aldrich, Germany), and images were captured with a Leica DM2000 microscope (Wetzlar, Germany) and digitally stored. Quantification of apoptotic nuclei and NF-κB positively labeled cells was performed by counting 400–500 cells in 20 microscopic fields.

### Immunoblotting

Western blot investigation of whole-cell lysates from HCT116 alginate beads was performed as described before (40). HCT116 cells were cultured in TME as described above and dissolved for 20 min in a sterile 55 mM sodium citrate solution to release the HCT116 cell from the alginate. After subsequent washing with Hank’s balanced salt solution, proteins were extracted on ice with lysis buffer [50 mM Tris/HCl, pH 7.2/150 mM NaCl/(v/v) Triton X-100/1 mM sodium orthovanadate/50 mM sodium pyrophosphate/100 mM sodium fluoride/4 µg/ml pepstatin A/1 mM phenylmethylsulfonyl fluoride (PMSF)] for 30 min. After centrifugation for 30 min at 10,000 *g* supernatant was stored at −80°C. Total protein content was measured with the bicinchoninic acid system (Uptima, France) using BSA as standard. Proteins were reduced with 2-mercaptoethanol and total protein concentrations adjusted (500 ng per lane total protein). Finally, proteins were separated by sodium dodecyl sulfate–polyacrylamide gel electrophoresis (SDS-PAGE), blotted onto a nitrocellulose membrane using a transblot apparatus (Bio-Rad, Munich) and, after blocking (skimmed milk powder 1% in PBS), primary antibodies (1:10,000) incubated overnight at 4°C. After additional washing, membranes were incubated for 1 h with secondary alkaline-phosphatase coupled antibodies (1:10,000), and specific binding was detected using nitro blue tetrazolium and 5-bromo-4-chloro-3-indoyl-phosphate (VWR, Germany). β-Actin was used to normalize samples to control, and densitometric quantification of bands was performed with the Quantity One program (Bio-Rad, Munich).

### Statistical Evaluation

All assays were performed three times as single assays using three different replicates. A Wilcoxon–Mann–Whitney test was used for statistical analysis. The results were presented as mean + SD or SEM and compared by one-, two-, or three-way ANOVA using SPSS Statistics if the normality test was passed (Kolmogorov–Smirnov test). A p-value of <0.05 was considered to detect statistically significant differences in the study.

## Results

The purpose of this study was to investigate the effect of Calebin A on 3D-alginate HCT116 CRC cells with an *in vitro* coculture of the multicellular TME (stromal components: MRC-5 fibroblast cells and T-lymphocytes) model (**Figures 1A, B**) to provide an *in vivo* approach of TME. This model helps to clarify the role of multicellular TME-induced inflammation and how these actions might be linked by specific signaling pathways during the initiation and development of human CRC.

### Calebin A Blocks CRC Cell Proliferation Promoted by Multicellular Proinflammatory TME

To examine the significance of inflammatory TME condition in CRC progression, we first investigated the proliferation capacity of HCT116 cells in 3D-alginate cultures alone (basal control) or co-cultured with MRC-5 fibroblasts and T-lymphocytes (multicellular proinflammatory TME cultures) and/or treated with TNF-β and/or with Calebin A (**Figures 1A, B**), by the MTT assay, as described in *Materials and Methods*. It has been previously shown that TNF-β as a proinflammatory cytokine promotes CRC cell malignancy (43). Therefore, to show that TNF-β is one of the most important proinflammatory cytokines produced by leukocytes, we also conducted a comparative experiment with TNF-β alone in parallel assays to lymphocytes.

**Figure 2:**
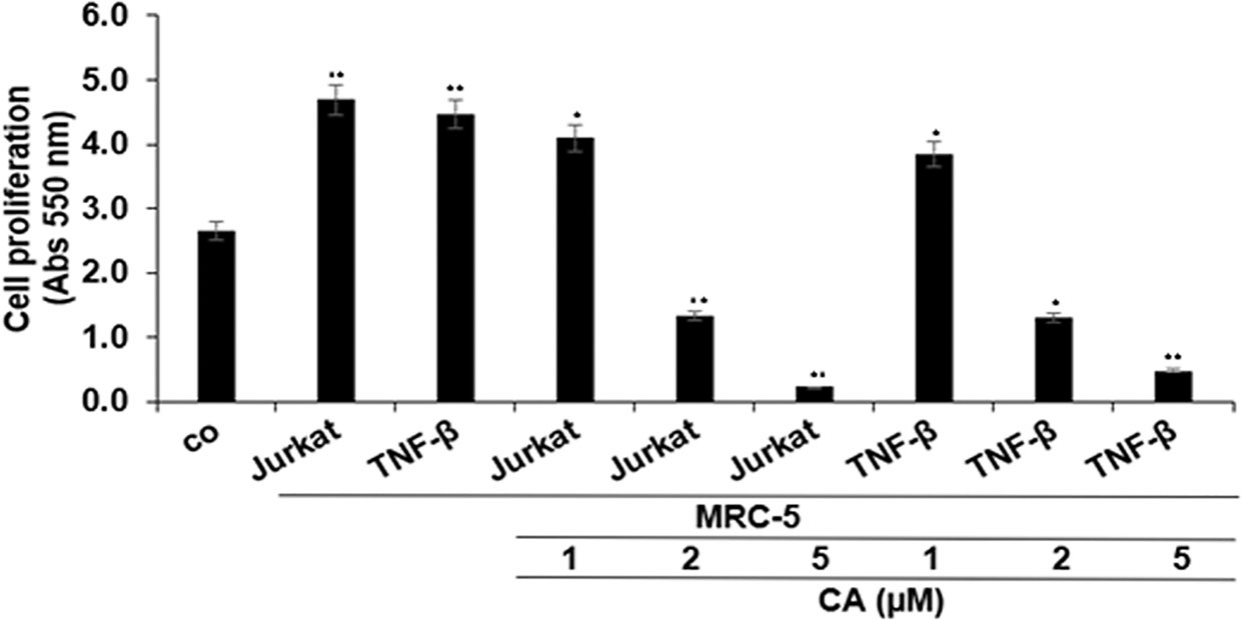
Effects of Calebin A on multicellular proinflammatory TME-induced HCT116 cell proliferation in 3D-alginate culture. Serum-starved cultures of HCT116 cells in 3D-alginate cultures alone (basal control = co) or co-cultured with fibroblasts and T-lymphocytes (proinflammatory multicellular TME) or co-cultured with fibroblasts and TNF-β (10 ng/ml) (TNF-β-TME) were either left untreated or treated with various concentrations of Calebin A (CA) (1, 2, and 5 µM) for 10 days in alginate cultures, and cell viability and proliferation were evaluated with the MTT assay, as described in *Materials and Methods*. All assays were performed with at least three independent replicates. *p < 0.05 and **p < 0.01 indicate a significant difference compared to the control group.

As shown in **Figure 2**, multicellular proinflammatory TME with T-lymphocytes or with TNF-β alone was found to significantly increase HCT116 cell proliferation, compared with basal control. On the contrary, cotreatment of these cells with Calebin A showed a marked dose-dependent decrease compared to the excess proliferation of CRC cells induced in both cases, and the maximum growth inhibition was observed in response to 5 µM Calebin A (**Figure 2**). This dose was selected for the subsequent investigations. Taken together, these results suggest that Calebin A indeed showed superior antitumor potency in a 3D-alginate stimulated multicellular proinflammatory TME.

### Calebin A Downregulates CRC Cell Colony Formation and Migration Promoted by Multicellular Proinflammatory TME

To examine that Calebin A mediates its antitumor actions in multicellular proinflammatory TME cultures through inhibition of colony formation and migration, the CRC cells were treated as described in detail in *Materials and Methods*, and the response was evaluated by phase-contrast light microscopy.

**Figure 3:**
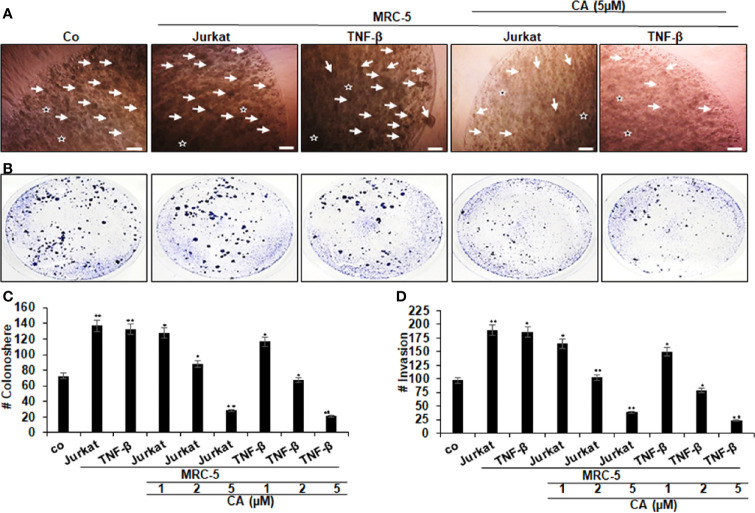
Effects of Calebin A on multicellular proinflammatory TME-induced HCT116 cell colony formation and invasion in 3D-alginate culture. Serum-depleted cultures of HCT116 cells in 3D-alginate (*) cultures alone (co) or co-cultured with fibroblasts (MRC-5) and T-lymphocytes (Jurkat) or cocultured with fibroblasts and TNF-β (10 ng/ml) were either left untreated or treated with different concentrations of Calebin A (CA) as described in *Materials and Methods*. **(A)** Colonosphere formation and **(B)** invasion were examined by light microscopy after 10 days. All experiments were performed at least three times. The number of colonospheres (A, arrows) was quantified by counting 20 different microscopic fields **(C)**, and the number of attached colonies as invasion parameter stained with toluidine blue was quantified in each well **(D)**. *p < 0.05 and **p < 0.01 indicate a significant difference compared with the control group. Magnification A: 24×, bars = 0.2 mm.

Additionally, HCT116 cells were investigated in alginate microenvironment cultures, without MRC-5 fibroblasts and T-lymphocytes as a basal control. As demonstrated in **Figure 3**, multicellular proinflammatory TME with T-lymphocytes or with TNF-β by itself prompted and significantly increased the number of colonosphere formations (**Figures 3A, C**) and invasion (**Figures 3B, D**) in HCT116 cells compared to that in basal control cultures, suggesting the pivotal role of multicellular proinflammatory TME cultures-mediated malignant potential of CRC cells. On the contrary, the concomitant treatment with Calebin A showed a dose-dependent inhibition of colonosphere formations (**Figures 3A, C**) and invasion (**Figures 3B, D**) in CRC cells in the TME co-cultures compared to that of control cultures. Taken together, these results underline that proinflammatory TME can stimulate malignancy of the tumor cells, and Calebin A exhibits anticancer and anti-invasive attributes in this multicellular proinflammatory TME.

### Calebin A Decreases Multicellular Proinflammatory TME-Induced Activation and Nuclear Translocation of p65-NF-κB in CRC Cells

**Figure 4:**
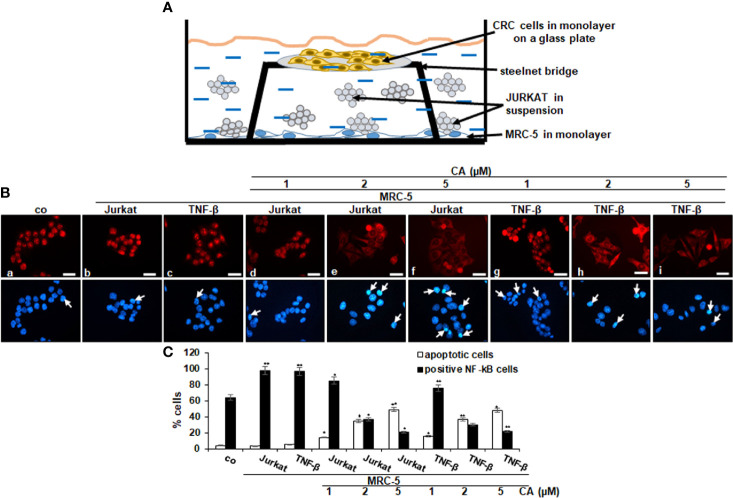
Effect of Calebin A on multicellular proinflammatory TME-induced activation and nuclear translocation of p65-NF-κB in CRC cells. **(A)** Schematic representing the experiment model of the HCT116 cells in TME for immunofluorescence assay. **(B)** Serum-starved HCT116 cells in monolayer cultures alone (co) or co-cultured with fibroblasts and T-lymphocytes or co-cultured with fibroblasts and TNF-β (10 ng/ml) were either left untreated, or treated with various concentrations of Calebin A (CA) (1, 2, and 5 µM), as described in *Materials and Methods*. Magnification 600×; scale bar = 30 mm. All experiments were performed at least in triplicate, and quantification of positively labeled p65-NF-κB-nuclei and apoptotic nuclei (white arrows) were performed by counting 400–500 cells from 20 different microscopic fields **(C)**. Values were compared with the control, and *p < 0.05 and **p < 0.01 were considered statistically significant.

It is known that proinflammatory cytokines promote tumor cell growth and invasion through activation of transcription factor NF-κB (18); therefore, we explored the expression and nuclear translocation of NF-κB associated with malignancy and survival of CRC cells and performed immunofluorescence labeling for p65-NF-κB as described in *Materials and Methods*. In the untreated control multicellular proinflammatory TME cultures, 98% of HCT116 cells revealed strong positive labeling for p65-NF-κB in the nucleus. A similar positive signal was observed in the TNF-β stimulated TME cultures (97%), while HCT116 cells as basal control showed 84% nuclear labeling (**Figures 4A–C**). Interestingly, treatment with Calebin A lowered the nuclear staining and nuclear translocation of p65-NF-κB in CRC cells in a dose-dependent manner (1, 2, and 5 µM) in both multicellular proinflammatory TME cultures (85%, 37%, and 21%) and in TNF-β-TME (76%, 30%, and 22%), respectively, suggesting the essential synergistic role of the paracrine interaction between HCT116, MRC-5 cells, and T-lymphocytes/TNF-β in maintaining tumor promotion. Taken together, these results suggest further that Calebin A modulates the tumorigenic impact promoted by proinflammatory cytokine TNF-β or T-lymphocytes, at least in part by the suppression of the NF-κB signaling pathway. Furthermore, we examined the extent of cell death by apoptosis using DAPI staining and fluorescence microscopy to understand the nuclear morphological changes that occurred during Calebin A treatment (**Figure 4C**). In the untreated multicellular proinflammatory- and TNF-β-TME cultures, HCT116 cells displayed usual nuclear size and minimal morphological changes resulting in 4% and 6% of the cells in apoptosis, similar to basal control cultures (4%). In contrast, a significant gain in fragmented nuclei and apoptotic morphological changes were observed in HCT116 cells co-treated with Calebin A in a dose-dependent manner with 14%, 35%, and 59% in multicellular proinflammatory TME co-cultures and with 16%, 37%, and 48% in TNF-β-TME co-cultures, respectively (**Figure 4C**). These results are in concordance with data from the MTT assay and support the fact that Calebin A significantly blocks protumorigenic effects of the proinflammatory TME in CRC cells by inducing apoptosis (**Figure 4C**).

### Calebin A Blocks Multicellular Proinflammatory TME-Induced NF-κB Activation and NF-κB-Regulated Gene End-Products in CRC Cells

**Figure 5:**
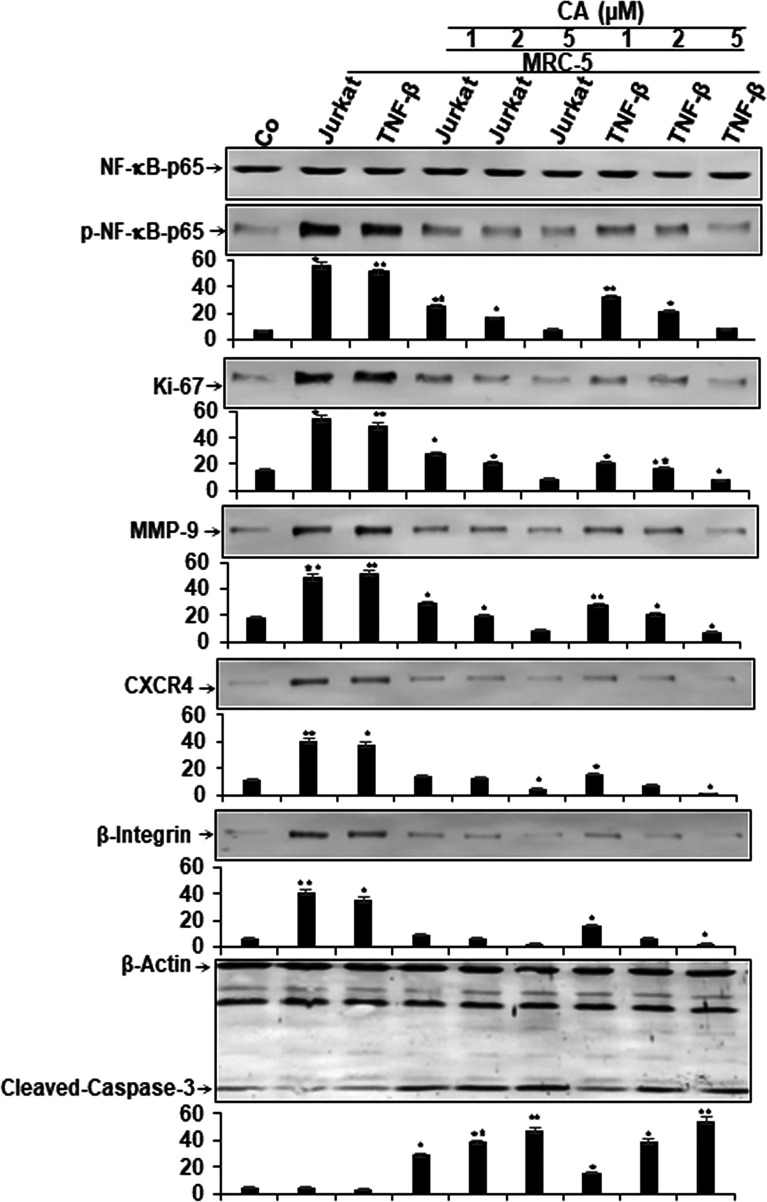
Effect of Calebin A on multicellular proinflammatory TME-induced activation of NF-κB and NF-κB-regulated gene end-products in CRC cells. Serum starved cultures of HCT116 cells in 3D-alginate cultures alone (co) or co-cultured with fibroblasts and T-lymphocytes or co-cultured with fibroblasts and TNF-β (10 ng/ml) were either left untreated or treated with various doses of Calebin A (CA) (1, 2, and 5 µM), as described in *Materials and Methods*. Immunoblotting of whole-cell lysates from HCT116 was performed for anti-p65-NF-kB, anti-phospho-p65-NF-κB, anti-β1-integrin, anti-MMP-9, anti-CXCR4, anti-Ki67, and anti-cleaved-caspase-3. β-Actin served as an internal loading control in all experiments. For densitometric evaluation, results are compared to control, and *p < 0.05 and **p < 0.01 were considered statistically significant.

Given that activation of the transcription factor NF-κB signaling cascade mediates the stimulation of the proinflammatory TME in CRC, we sought to examine if Calebin A had the potential in inhibiting TME-promoted NF-κB activation and NF-κB-promoted gene products involved in proliferation (Ki-67), invasion (MMP-9), metastasis (CXCR4, β1-integrin), and apoptosis (cleavage of Caspase-3). The multicellular TME cultures were treated or either left untreated as described in *Materials and Methods*. Additionally, HCT116 cells alone in the alginate microenvironment were used as basal control. To evaluate whether Calebin A inhibits the TME-induced activation of NF-κB, HCT116 were examined for the phosphorylated form of the p65-NF-κB subunits. The results demonstrated that significantly more activation of p65 subunit in multicellular proinflammatory- and TNF-β-TME compared to basal control HCT116 alginate cultures. Calebin A significantly inhibited the multicellular proinflammatory- or TNF-β-TME-induced phosphorylation of p65 subunits in a concentration-dependent manner in HCT116 cells. Furthermore, the expression of mentioned NF-κB-promoted gene products was markedly upregulated in the multicellular proinflammatory TME and TNF-β-TME cultures compared to basal control HCT116 alginate cultures (**Figure 5**). Calebin A treatment in these cultures downregulated the expression of mentioned NF-κB-promoted gene products in a concentration-dependent manner (**Figure 5**). Densitometric analysis of Western blot experiments confirmed dose dependently the downregulation of NF-κB, Ki-67, MMP-9, CXCR4, and β1-integrin, and upregulation of apoptosis (cleavage of caspase-3) in HCT116 cells in TME cultures treated with Calebin A (**Figure 5**). These results are in agreement with the previous reports that suggest that proinflammatory agents are upregulated in the TME (44–46) and are regulated by proinflammatory transcription factor NF-κB (47). In addition, it is in concordance with the suppression of p65-NF-κB observed through immunofluorescence analysis in the current study, and with the existing literature that describes the potential of Calebin A in proinflammatory monocellular-TME culture (39). Taken together, these findings underline that the multicellular proinflammatory TME promotes HCT116 tumor cell progression, at least in part through NF-κB signaling pathways, and this pathway could be specifically inhibited by Calebin A. Thus, Calebin-A-mediated regulation of the NF-κB signaling pathway might exert an antitumorigenic effect in CRC tumor.

### Cancer Stem Cells Are Targeted in Multicellular Proinflammatory TME Co-Cultures by Calebin A

**Figure 6:**
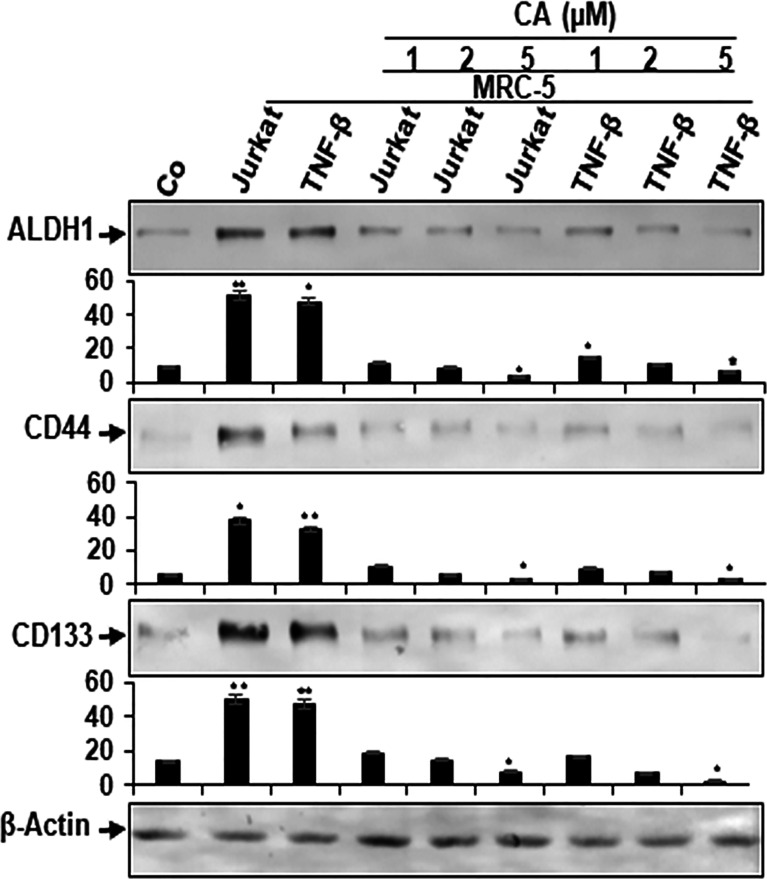
Effect of Calebin A on multicellular proinflammatory TME-induced activation of cancer stem cells. Serum-starved cultures of HCT116 cells in 3D-alginate cultures alone (basal control = co) or co-cultured with fibroblasts and T-lymphocytes (proinflammatory multicellular TME cultures) or co-cultured with fibroblasts and TNF-β (10 ng/ml) (TNF-β-TME) were either left untreated or treated with various concentrations of Calebin A (CA) (1, 2, and 5 µM), as described in *Materials and Methods*. Immunoblotting of whole-cell lysates from HCT116 cells was performed for anti-CD133, anti-CD44, and anti-ALDH1 in HCT116 cells. β-Actin served as an internal loading control in all experiments. Densitometric values were compared with the control, and *p < 0.05 and **p < 0.01 were considered statistically significant.

It has been previously reported that the TME plays an important role in the induction of the cancer stem cells (CSCs) affected by stroma, inflammatory cells, cytokines, and growth factors secreted by the stromal fibroblasts (48, 49). Therefore, we investigated the reaction of CSCs within the CRC cell population, and multicellular proinflammatory TMEs of HCT116 cells were either left untreated or treated as described in *Materials and Methods*. In addition, HCT116 cells alone in the alginate microenvironment were used as basal control for these experiments. We examined CSC markers (CD133, CD44, and ALDH1) expression for tumor formation capacity and the potential effect of Calebin A on these CSC markers. Control alginate microenvironment cultures (without MRC-5 fibroblasts or T-lymphocytes) of HCT116 cells showed basal expression of CSC markers (**Figure 6**). Contrary to this, the immunoblotting investigation showed notable upregulated levels of CD133, CD44, and ALDH1 in HCT116 cells from multicellular and TNF-β-TME cultures (**Figure 6**). However, treatment with Calebin A significantly downregulated CSC marker expression in a concentration-dependent manner, suggesting the prominent targeting effect of Calebin A on CSCs (**Figure 6**). Densitometric analysis of Western blot experiments showed and confirmed the above-mentioned results (**Figure 6**). Altogether, these results suggest that the paracrine interaction between tumor, stromal cells, and T-lymphocytes in 3D-alginate multicellular proinflammatory TME cultures are essential in directing CSCs phenotype. These findings further underline the essential role of Calebin A in modulating multicellular proinflammatory-, similar to TNF-β-TME-enhanced and stimulated CSCs. Moreover, these results further suggest that the antitumorigenic effects of Calebin A are, in part, mediated through the downregulation of the CSCs pathway and also the through inhibition of NF-κB activation.

## Discussion

The modulation of tumor and stromal cells in the TME is crucial for tumor cell progression and malignancy. To better understand the underlying paracellular interaction between different cells in TME, we have created a new 3D-alginate multicellular proinflammatory TME model (**Figures 1A, B**) composed of tumor cells, stromal fibroblasts cells, and T-lymphocytes that better mimics the *in vivo* heterogeneous proinflammatory TME. In the present study, we have shown how Calebin A (a component of *Curcuma longa*), at least partially, modulates the inflammation-promoted survival and progression of CRC cells.

The results of this study indicated that Calebin A blocked the proliferation, migration, and vitality of HCT116 cells in 3D-alignate multicellular proinflammatory TME culture and inhibited the activation of the transcription factor p65-NF-κB and its nuclear translocation. Calebin A was found to significantly suppressed NF-κB-promoted gene products associated with cell survival, proliferation, and metastasis and induced apoptosis in multicellular proinflammatory TME cocultured with T-lymphocyte cultures, and TNF-β induced TME. In addition, Calebin A targeted and inhibited CSCs of the CRC population in the multicellular proinflammatory TME cultures and TNF-β-TME. To our knowledge, we have shown for the first time that the cytokine TNF-β-TME mediates proinflammatory signals in alginate HCT116 3D-alginate, similar to T-lymphocytes multicellular TME, which underlines the important role of this cytokine in the proinflammatory progression of CRC cells.

*In vitro* 3D-TME models have become an indispensable asset in the evaluation of tumor cell dynamics before *in vivo* research. These models are recognized as intermediatory between *in vitro* cancer cell cultures and *in vivo* investigation (40, 50–55). The 3D-TME models have gained increasing popularity in tumor cell research, preclinical tumor research, and drug discovery because these models recapitulate the essential properties of *in vivo* tumor conditions (e.g., cell adhesion, crosstalk, microenvironment, and necrosis) that cannot be reproduced in conventional 2D-tumor cultures in monolayers. Due to the lack of a tumor-specific architecture in 2D-tumor models, the cells represent a decreased malignant phenotype compared to the tumor cells of *in vivo* settings (54, 56, 57). For these reasons, the results of 2D-cultures obtained in *in vitro* conditions often cannot be reproduced in *in vivo* conditions.

Increasing experimental evidence over the past decades has implicated chronic inflammation as a significant and important trigger mechanism for the development of various cancers (58, 59). Previous studies have also reported that chronic inflammation with a low tumor grade modulates the intricate cross-talk between tumor cells and their immediate microenvironment, thereby dramatically increasing the malignancy potential of tumors (20, 60). In fact, environmental stress factors are responsible for 95% of cancers incidence because they mediate and trigger chronic inflammation in patients (20). Interestingly, several studies suggested that proinflammatory cytokines such as members of the TNF family play a key role in cancer initiation and progression (60, 61). Furthermore, in studies reported by our laboratory and others as well, it has already been shown that the proinflammatory cytokine TNF-β promotes tumor progression and thus activates CRC cells malignancy (HCT116 cells and HCT116 5-FU resistant cells) with the same efficacy as TNF-α (23, 62, 63). These data highlight an essential role of TNF-β in the inflammatory environment and in the stimulation of the ongoing TME interactions. In addition, it has been reported that TNF-β produced by tumor-associated lymphocytes in the TME induces angiogenesis by signaling *via* the canonical NF-κB signaling pathway (26). In addition, autocrine signaling of TNF-β was shown to promote disease progression by maintaining NF-κB phosphorylation in Hodgkin’s lymphoma (63).

In order to investigate and better establish the role of the proinflammatory cytokine TNF-β in inflammation-induced TME in CRC, we conducted comparative experiments between TME with T-lymphocytes and TME with TNF-β. Moreover, it is known that the inflammation and associated with an upregulation of proinflammatory mediators such as IL-1β, IL-6, IL-8, and TNF-α, and prostaglandins by leukocytes play a major role in tumor development in TME (64–67). In addition, several studies from other laboratories including our own have previously reported that Calebin A shows significant anti-inflammatory effects in the migration, proliferation, and invasion of tumor cells (23, 35, 37). Furthermore, in accordance with our recently published findings, Calebin A has been demonstrated to inhibit TNF-β-induced survival, invasion, and antiapoptotic capacity in CRC cells *in vitro* (23, 39, 68). However, in the current work, we have shown for the first time the suppression of proliferation, survival, and migration of CRC cells by Calebin A in a multicellular proinflammatory TME HCT116 3D-alginate culture mimicking physiological TME.

We have revealed that Calebin A alone significantly induced apoptosis in the multicellular proinflammatory TME of HCT116, similar to TNF-β-TME, probably by suppressing the activation, phosphorylation of NF-κB and its translocation in the nucleus in HCT116 cells. Suppression of phosphorylation and activity of NF-κB subunits could be a potential therapy and chemopreventive intervention in colon carcinogenesis by Calebin A. To support this assumption, in previous studies, Calebin A treatment was found to sensitize human lung, cervical cancer, and CRC cells to paclitaxel or cisplatin or 5-FU effectively inhibiting the NF-κB pathway (23, 37, 39). We also found that Calebin A suppresses the expression of gene products associated with cell proliferation (Ki-67), invasion (MMP-9), metastasis (CXCR4, integrin β1), and apoptosis (Caspase-3) in HCT116 multicellular proinflammatory TME and TNF-β-TME. Since these tumorigenic proteins are promoted by NF-κB, it is likely that the suppression of NF-κB contributes to the downmodulation of these proteins. These data suggest that Calebin A may be able to act as an active multitargeting anticancer agent, and its action is partially mediated by the suppression of NF-κB and NF-κB-induced gene products in CRC-TME cells.

Our results in this study showed that T-lymphocytes activated NF-κB and NF-κB-promoted proteins in HCT116 CRC cells in multicellular TME cultures, similar to the proinflammatory cytokine TNF-β-TME. Furthermore, it is known that NF-κB is stimulated by different cytokines or growth factors in different tumor cells (69, 70). In the past, TNF-β, a member of the TNF family, has been reported to trigger inflammatory effects in CRC cells with a potency similar to TNF-α (25, 62). We have also previously reported that TNF-β promoted NF-κB activation, and this was suppressed by other natural substances such as resveratrol or curcumin (25, 40, 62). Recently, our group reported that TNF-β stimulates the NF-κB signaling pathway and NF-κB-induced gene products in CRC tumor cells, which was associated with survival, proliferation, invasion, and epithelial-to-mesenchymal transition of tumor cells (25, 62), similar to the results of multicellular proinflammatory TME in the current study. These results underline that the cytokine TNF-β, like TNF-α, is one of the most important cytokines in inflammatory multicellular TMEs that are produced by T-lymphocytes (67).

In order to better understand the mechanism involved in the survival and proliferation of tumor cells in the TME, it might be important to identify other types of new targets for the chemopreventive and chemotherapeutic agent Calebin A. In the current study, we have also found for the first time that the expression of cancer stem cells (CSC) in the HCT116 cell population was significantly increased in multicellular pro-inflammatory-, similar to TNF-β-TME cultures, compared to HCT116 alginate basic control cultures. These results dictate that multicellular pro-inflammatory TME is a fundamental factor that induces and supports the formation of CSCs subpopulations. Furthermore, it has been reported in the literature earlier that CSCs are responsible for the onset, aggressiveness, progress, and resistance of the cancer population (71, 72). Interestingly, Calebin A was shown to inhibited the expression of various stem cell markers (CD44, CD133, and ALDH1) in HCT116 multicellular proinflammatory TME and TNF-β-TME cultures. Accumulating evidence has reported that CSCs are one of the main reasons for therapy resistance, therapy failure, and tumor recurrence (73–75), which underlines the potentially important role of Calebin A in preventing tumor cell proliferation, invasion, metastasis, and induction of apoptosis in tumor cells. These results suggest that when designing new approaches in cancer treatment, attention should be paid to the specific target the interactions TME and CSCs. These data are consistent with results from other studies suggesting that other natural agents such as curcumin and resveratrol significantly reduced the expression of CSC biomarkers in CRC cells (24, 76). Overall, the modulation of interaction in multicellular proinflammatory TME, similar to TNF-β-TME cultures by Calebin A, could be a novel therapeutic approach to targeting inflammation during CRC pathogenesis.

In conclusion, most malignant tumors, including colon cancer, represent very dynamic structures that create new tumor cell with morphological and genotypical changes within the tumor mass. Such malignancies present a highly variable sensitivity to therapeutics and incur resistance to the treatment. In combinatorial approaches involving conventional therapies with plant-derived molecules with proven cytotoxic, multitargeted effects should be superior compared to cancer monotherapy and may delay the development of drug resistance in cancer (77). Therefore, further research warrants the need for the identification of new molecules (including plant-derived compounds) with well-validated anticancer properties for further progressing the novel and more efficacious therapies within the clinical oncology. Such multicellular-TME-based models and investigations are crucial for their heterogenicity similar to those under *in vivo* conditions, and this is pivotal for the discovery, screening, and testing of new, promising anticancer drugs and strengthening preclinical and clinical research. The critical role of the multicellular TME in controlling tumor progression and metastasis is now widely recognized. Hence, the targeted attack on the formation of a specialized microenvironment, and thus the inhibition of the survival and growth of tumor cells or CSCs by a multitargeted drug such as Calebin A, is therefore of great clinical relevance in the future of cancer medicine.

## Data Availability Statement

The raw data supporting the conclusions of this article will be made available by the authors, without undue reservation.

## Author Contributions

CB, AK, MSh performed all the different experiments and analyses. AK, AK, CB, BA, PK, MSa, and MSh were responsible for the study design, data interpretation, revised the paper, and all authors approved the final version of the manuscript. MSh provided expert assistance and supervised the preparation of the manuscript. All authors contributed to the article and approved the submitted version.

## Conflict of Interest

The authors declare that the research was conducted in the absence of any commercial or financial relationships that could be construed as a potential conflict of interest.

## Publisher’s Note

All claims expressed in this article are solely those of the authors and do not necessarily represent those of their affiliated organizations, or those of the publisher, the editors and the reviewers. Any product that may be evaluated in this article, or claim that may be made by its manufacturer, is not guaranteed or endorsed by the publisher.
